# Diagnosis of *Tropheryma whipplei* pneumonia using targeted nanopore sequencing: a rare case report and literature review

**DOI:** 10.3389/fmed.2025.1556735

**Published:** 2025-04-30

**Authors:** Miao Yu, Wenbin Wang, Wei Chen, Kexin Ye, Yuqing Zhou, Deep K. Vaishnani, Chen Chen, Xuancheng Jin, Xiaojun Zhu, Jun Ma, Fengfei Qian, Xiaohua Zhong, Chuanhua Nie

**Affiliations:** ^1^Pulmonary and Critical Care Medicine, Hangzhou Linping District Hospital of Integrated Traditional Chinese and Western Medicine, Hangzhou, Zhejiang, China; ^2^Renji College, Wenzhou Medical University, Wenzhou, Zhejiang, China; ^3^School of International Studies, Wenzhou Medical University, Wenzhou, Zhejiang, China; ^4^School of the First Clinical Medical Sciences (School of Information and Engineering), Wenzhou Medical University, Wenzhou, Zhejiang, China; ^5^Department of Pathology, Ruian People’s Hospital, Wenzhou, Zhejiang, China; ^6^Department of Pathology, First Affiliated Hospital of Wenzhou Medical University, Wenzhou, Zhejiang, China

**Keywords:** *Tropheryma whipplei*, pneumonia, tNGS, nanopore sequencing, BALF, case report

## Abstract

*Tropheryma whipplei* (TW) pneumonia is a rare disease caused by *Tropheryma whipplei* infection. Due to the difficulty in obtaining pathogenetic evidence, the misdiagnosis rate is high, posing significant challenges for early diagnosis and treatment. Targeted sequencing technology based on nanopore sequencing technology (tNGS) allows for detecting rare pathogens that are difficult to identify using traditional methods, leading to improved detection rates for TW. This paper reports a case of pneumonia diagnosed as a TW infection through the use of tNGS on bronchoalveolar lavage fluid (BALF), with the patient showing improvement after treatment with sequential administration of meropenem followed by compounded sulfamethoxazole.

## 1 Introduction

*Tropheryma whipplei* (TW) is the causative agent of Whipple’s disease (WD), characterized by various clinical symptoms, including diarrhea, weight loss, lymphadenopathy, and polyarthritis (1). TW can also affect the nervous, cardiovascular, and respiratory systems, resulting in conditions such as pneumonia, with symptoms including fever, cough, and chest pain. Although gastrointestinal involvement is common, pulmonary involvement is rare but can be life-threatening if not diagnosed and treated promptly. WD is a rare multi-systemic disorder with an incidence rate of approximately 1 in 1,000,000 individuals (2).

This paper reports a case of a patient with chronic anemia, primarily presenting with gastrointestinal symptoms, who developed extensive pneumonia. The diagnosis of *Tropheryma whipplei* infection was confirmed using tNGS. Additionally, a literature review was conducted to provide clinicians with diagnostic and therapeutic insights, aiming to enhance the clinical management of this rare disease.

## 2 Case description

The patient, a 66-year-old male, was admitted to Hangzhou Linping District Hospital of Integrated Traditional Chinese and Western Medicine with a primary complaint of “upper abdominal discomfort accompanied by nausea for half a month.” He had a history of heavy vomiting following alcohol intoxication about 2 weeks prior, after which he gradually developed symptoms of upper abdominal discomfort and bloating, which worsened after meals, along with nausea and intermittent abdominal pain. The abdominal discomfort was relieved after bowel movements, which were loose, irregular, and unformed, with varying frequency (sometimes 2–3 days without a bowel movement, other times 2–3 times per day). He reported feeling feverish during abdominal pain episodes, though no specific temperature was recorded. He also experienced fatigue and poor appetite and noted a weight loss of approximately 5 kg over the past 2 weeks.

The patient’s medical history included mild anemia for over 10 years without specific treatment. He had a diagnosis of prostatic hyperplasia and thyroid nodules 3 years ago, with no long-term medication. He denied having a history of hypertension, coronary artery disease, diabetes, or rheumatoid arthritis. Socially, the patient had a long-term smoking and drinking habit, consuming about 20 cigarettes and 100 mL of liquor daily for over 20 years. He denied any history of exposure to contaminated water or unsanitary environments.

Upon admission, the patient’s physical examination showed a temperature of 36.2°C, a pulse of 86 beats per minute, a respiration rate of 19 breaths per minute, and a blood pressure of 107/61 mmHg. He was conscious and alert with a soft demeanor, and there were no signs of cyanosis of the lips or palpable lymphadenopathy. Respiratory auscultation revealed coarse breath sounds bilaterally, with slight moist rales noted in the left lower lung. A cardiac examination showed a regular rhythm without pathological murmurs. The abdomen was soft, with no discernible or rebound tenderness, and there was no evidence of hepatosplenomegaly or peripheral edema.

## 3 Therapeutic process

On admission to the respiratory department, the patient presented with a maximum temperature of 38.1°C and an oxygen saturation of 94%, prompting oxygen therapy. The treatment regimen included levofloxacin at 0.5 g once daily, combined with cefoperazone-sulbactam at 3 g every 8 h, alongside anti-acid medications for gastric protection and fluid supplementation.

The following day, the patient’s temperature returned to normal, with only a slight cough present—no sputum production, chest tightness, chest pain, or difficulty breathing were reported. However, gastrointestinal symptoms, including abdominal distension, pain, and stool consistency and frequency, showed no significant improvement.

On the fourth day of hospitalization, the patient experienced fever again, with a maximum temperature of 37.6°C, which self-resolved to within the normal range. A follow-up complete blood count revealed a white blood cell count of 4.8 × 10^9^/L and a neutrophil count of 4.1 × 10^9^/L, with a highly sensitive C-reactive protein level of 5.1 mg/L. Given these findings, pneumonia due to heat absorption was considered, and the original treatment plan was continued. Subsequently, the patient experienced daily fevers, with a maximum temperature of 38°C, which also self-resolved to normal. Digestive symptoms remained similar, with no exacerbation of the cough or respiratory symptoms such as chest tightness or difficulty breathing.

**Figure 1:**
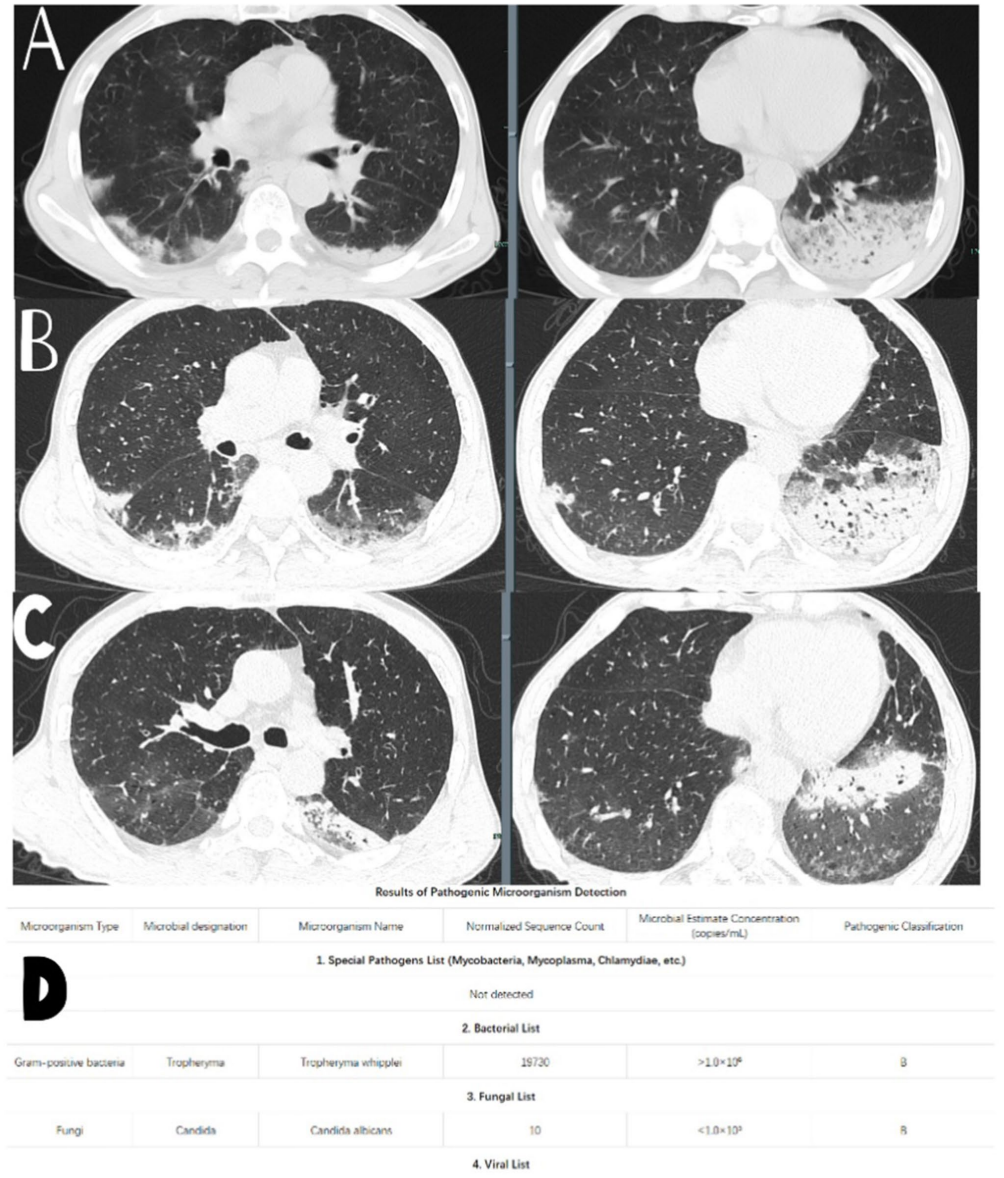
**(A)** Chest CT performance when the patient was admitted to the hospital. **(B)** Chest CT performance on the 7th day of admission. **(D)** Test results of the tNGS. **(C)** Chest CT performance after 1 month of regular treatment of the patient.

On the seventh day of hospitalization, a repeat complete blood count showed a white blood cell count of 5.6 × 10^9^/L, a neutrophil count of 4.2 × 10^9^/L, and a highly sensitive C-reactive protein level of 44.7 mg/L. Total protein was measured at 28 g/L, and total IgE was 375.7 IU/mL. A follow-up chest CT scan (see Figure 1B) indicated persistent lung infections with no significant improvement compared to the previous scan, alongside a small amount of left pleural effusion.

A bronchoscopy was performed the next day, and bacterial cultures from bronchoalveolar lavage fluid showed no bacterial growth. However, tNGS from the bronchoalveolar lavage indicated the presence of Whipple’s disease (sequence number 19730, estimated microbial concentration >1.0 × 10^6^) and *Candida albicans* (sequence number 10, estimated microbial concentration <1.0 × 10^3^) as shown in Figure 1D. The patient had a history of long-term anemia and a previous syphilis infection, along with underlying immunocompromised status and a history of high-risk aspiration, raising the suspicion of opportunistic infections. Therefore, the primary diagnosis considered was “aspiration pneumonia (TW infection).”

Consequently, on the eighth day of hospitalization, levofloxacin and cefoperazone-sulbactam were discontinued and replaced with meropenem at 1 g every 8 h and methylprednisolone at 8 mg twice daily.

On the tenth day of hospitalization, the patient’s temperature returned to the normal range, with significant improvement in appetite, reduced abdominal distension and pain, and improved stool consistency. Considering the effectiveness of the treatment plan, the same regimen continued, with a gradual tapering of methylprednisolone, which was stopped on the twelfth day of hospitalization.

By the thirteenth day, the patient’s gastrointestinal symptoms had largely resolved, and no further fevers were recorded. During this period, a gastrointestinal endoscopy indicated chronic non-atrophic gastritis and colonic polyps, with a duodenal biopsy showing negative results on periodic acid-Schiff staining.

Repeat tests for complete blood count, C-reactive protein, liver and kidney function, and electrolytes remained generally normal. The patient was considered stable enough for discharge, with instructions to continue oral sulfamethoxazole and trimethoprim at 2 tablets every 12 h for maintenance treatment. The patient was advised to follow up with phone calls or visits in the short term after discharge, and no recurrence of symptoms was observed post-discharge.

After a month of treatment, a repeat chest CT scan (see Figure 1C) showed partial resolution of the lung infection.

## 4 Diagnostic evaluation

The patient was admitted to the Gastroenterology Department with a preliminary diagnosis of “chronic gastritis” for further evaluation and treatment.

Auxiliary examinations revealed the following results: a white blood cell count of 3.8 × 10^9^/L and a neutrophil count of 2.9 × 10^9^/L. High-sensitivity C-reactive protein (hs-CRP) was measured at 57.6 mg/L, and interleukin-6 (IL-6) was 46.2 pg/mL. Total protein level was 31 g/L, and creatinine was 53 μmol/L. D-dimer was measured at 1040 ng/mL. The syphilis antibody test was positive (with a repeat TRUST test returning negative). Total IgE levels were at 1571.6 IU/mL. Coagulation function, thyroid function, liver and kidney function, electrolytes, brain natriuretic peptide, autoantibodies, and rheumatoid immunological factors were all essentially normal.

Chest, abdominal, and pelvic CT scans indicated bilateral lung infections and prostatic hyperplasia (see Figure 1A). A consultation with the Respiratory Department was requested, and considering the patient’s history and current laboratory findings, the following diagnoses were considered: (1) Pneumonia (aspiration being the primary concern); (2) Chronic gastritis; (3) Anemia; (4) Thyroid nodules; and (5) Prostatic hyperplasia. A transfer to the Respiratory Department for further diagnosis and treatment was recommended.

## 5 Discussion

Whipple’s disease is a rare systemic disorder, with patients commonly presenting with symptoms such as weight loss, arthralgia, diarrhea, and abdominal pain (2, 3). Due to its low incidence, epidemiological statistics for classical Whipple’s disease are challenging to obtain, but existing evidence suggests an estimated prevalence of approximately 1 in 1,000,000, with an annual incidence of about 1 to 6 cases per 10 million people (2). Although the incidence of typical Whipple’s disease is very low, *Tropheryma whipplei*, the causative agent, is now considered a widely distributed bacterium, with prevalence rates in specific populations, potentially reaching 12–25%, such as in wastewater treatment workers, HIV-infected individuals, and the homeless. Additionally, family members of patients with chronic Whipple’s disease are also at a higher risk of carrying this bacterium, indicating that it is primarily transmitted via the fecal-oral route (4). The clinical symptoms of Whipple’s disease are often diverse, with the gastrointestinal tract being the most frequently affected, leading to abdominal pain, diarrhea, poor appetite, and weight loss. The disease can also affect joints, causing arthritis or joint pain (5). *Tropheryma whipplei* can also involve the nervous system, which is one of the most severe complications, as the involvement of the central nervous system (CNS) has a high mortality rate (4). Cardiac involvement can lead to endocarditis (5). Pulmonary involvement is relatively rare but can sometimes be prominent, typically presenting symptoms such as fever, chest pain, and cough. Chest CT may reveal nodular lesions, inflammatory changes, and pleural effusion (6).

The diagnosis of Whipple’s disease (WD) faces significant challenges due to its broad and nonspecific clinical manifestations and its extremely low incidence, often leading to a diagnosis in the later stages of the disease (7). Currently, the most common diagnostic methods are tissue pathological biopsy and PCR. These tests are generally straightforward to perform, while the culture of *Tropheryma whipplei* presents specific difficulties, and most laboratories cannot carry it out. Thus, it is not a routine testing option. Since classical WD most frequently involves the gastrointestinal tract, a large number of classical WD patients typically have a significant bacterial presence in the duodenal mucosa. The duodenal mucosa often shows dilated villi and ectopic lymphatics, appearing pale yellow (4, 8).

In diagnosing classical WD, the foam cells in the affected tissue (primarily the small intestine) may show a positive result for PAS staining, as these macrophages contain PAS-positive granules resistant to amylase but negative for Ziehl-Neelsen staining. However, in patients without gastrointestinal symptoms, PAS staining of duodenal biopsies may yield negative results (9). Although PAS staining is frequently used clinically, it is not a highly specific method for diagnosing Whipple’s disease, as PAS-positive foam cells can also be found in patients infected with other bacteria, such as *M. avium*, *Mycobacterium intracellulare*, Bacteroides, *Bacillus cereus*, Blastomyces, or fungi (10).

PCR technology has gradually become the mainstream method for diagnosing WD due to its high specificity and sensitivity (5). A study comparing the application of PCR technology for the differential diagnosis of patients with Whipple’s disease and carriers indicated that PCR offers greater specificity and sensitivity than PAS staining of biopsy samples. The high specificity and low CT values of PCR from duodenal biopsy tissues may be more helpful in diagnosing WD, especially in patients whose biopsy specimens show negative PAS staining (11).

In patients with pneumonia caused by *Tropheryma whipplei* (TW) infection, clinical symptoms often lack specificity compared to typical bacterial pneumonia. Chest CT findings are also nonspecific, and traditional laboratory tests rarely provide direct evidence of TW infection. Due to the rarity of the pathogenicity of TW, clinicians often lack diagnostic experience, which can lead to misdiagnosis and missed diagnoses of the disease. Therefore, obtaining BALF for PCR testing during bronchoscopy in pneumonia patients is highly significant. Compared to metagenomic next-generation sequencing (mNGS), tNGS employs a screening process with specific library preparation and microbial sequencing libraries (12). This approach reduces costs and significantly shortens the detection time while covering over 800 types of microorganisms, including bacteria, viruses, and fungi.

In recent years, there has been a slight increase in the number of cases diagnosed with pneumonia caused by *Tropheryma whipplei* (TW) infection globally using mNGS. However, cases diagnosed using tNGS remain exceedingly rare. This patient had a long history of anemia and a prior history of syphilis, indicating the possibility of opportunistic infections due to immune dysfunction. Additionally, the patient had a history of heavy vomiting due to alcohol intoxication prior to the onset of symptoms, which may have increased the risk of aspiration and, consequently, of TW infection.

During the first week of treatment, the combination of levofloxacin and cefoperazone-sulbactam proved ineffective against the infection. Subsequently, bronchoscopy with BALF was performed, revealing a high sequence count of TW via tNGS. The treatment was then switched to meropenem for 2 weeks, significantly resolving the patient’s clinical symptoms and lung lesions.

Considering the patient’s clinical presentation, laboratory results, and response to antimicrobial treatment, it was concluded that the pneumonia was caused by TW infection. Furthermore, as the endoscopic examination to obtain duodenal biopsy samples was performed only after 1 week of meropenem treatment, there may have been significant interference with the PAS staining results, potentially leading to false-negative outcomes. Given the patient’s initial symptoms of abdominal pain, diarrhea, and weight loss, TW’s involvement in the gastrointestinal tract should still be considered. The final diagnosis was pneumonia due to TW infection, but the possibility of Whipple’s disease could not be ruled out.

There is no standard treatment protocol for Whipple’s disease, nor are there any relevant domestic or international guidelines or expert consensus. All treatment options in use are empirical. Although antibiotic therapy for *Tropheryma whipplei* infection typically leads to rapid clinical improvement, eradicating the bacterium requires prolonged treatment (3, 4). Symptoms such as diarrhea, joint pain, and fever usually resolve within a week, while other symptoms may take several weeks to disappear (3, 4, 13). Patients with advanced symptoms involving the eyes, heart, and nervous system are often difficult to cure, and these patients tend to have high rates of recurrence and mortality (3, 4, 14). Some patients with early symptoms also find treatment challenging, potentially entering a state of lifelong infection. The reasons for this include the possibility of bacteria persisting permanently in the host, the development of antibiotic resistance, and the risk of reinfection (15).

Since the first successful treatment of Whipple’s disease in 1952, several antibiotic combinations have been used, including azithromycin, streptomycin, tetracycline, ceftriaxone, meropenem, trimethoprim-sulfamethoxazole, doxycycline, and hydroxychloroquine (4, 16, 17). Tetracycline has a high recurrence rate, particularly concerning neurological relapses, which has led to a preference for antibiotics that effectively penetrate the blood–brain barrier, such as sulfonamides (18).

The current standard treatment regimen consists of ceftriaxone 2 g once daily or meropenem 1 g every 8 h for 14 days, followed by oral trimethoprim-sulfamethoxazole for 12 months (4, 16). However, *in vitro* data sequence analysis indicates that *Tropheryma whipplei* lacks the target for trimethoprim (dihydrofolate reductase), suggesting resistance to this antibiotic. For this reason, doxycycline is recommended (16, 17, 19); this regimen is also suitable for patients who cannot tolerate trimethoprim-sulfamethoxazole. When using alternative oral treatment regimens to trimethoprim-sulfamethoxazole, a combination of doxycycline 200 mg daily and hydroxychloroquine 600 mg daily for 12 months is preferred (17, 20).

It is worth noting that the recurrence rate of this disease is high; thus, in cases of frequent recurrence, the aforementioned alternative regimen (doxycycline 200 mg daily combined with hydroxychloroquine 600 mg daily for 12 months) should be followed by lifelong treatment with doxycycline to prevent reinfection (17, 20, 21). Of course, for patients requiring lifelong therapy, it is crucial to pay closer attention to potential adverse drug reactions and the development of antibiotic resistance, including retinal toxicity and skin manifestations (22).

## 6 Conclusion

In summary, pneumonia caused by *Tropheryma whipplei* (TW) infection is rare, with gastrointestinal symptoms being the most prominent manifestation. However, TW pneumonia without any respiratory symptoms is even rarer. Due to the nonspecific clinical presentations, routine laboratory tests rarely provide evidence of TW infection, and the Periodic Acid-Schiff (PAS) staining has a high false-positive rate, which can lead to missed or misdiagnosed cases and subsequently delay treatment.

With the rapid advancement of diagnostic technologies, the gradual maturation of PCR techniques, and the advantages of tNGS, including cost-effectiveness, shorter detection time, and high sensitivity, the early diagnosis rate of TW pneumonia has significantly improved. This facilitates quicker and more accurate diagnosis, leading to more effective treatment options and ultimately improving patient outcomes while shortening the duration of therapy. Therefore, in routine clinical practice, for patients with severe pulmonary infections of unknown etiology, it is advisable to conduct tNGS testing as soon as conditions permit and adjust treatment plans based on the results. Furthermore, due to the scarcity of clinical cases and a lack of data, standard treatment protocols remain to be further explored, and the prognosis of the disease requires continued observation and statistical analysis.

## Data Availability

The original contributions presented in the study are included in the article/supplementary material, further inquiries can be directed to the corresponding author.
